# Cooled Radiofrequency at Five Revised Targets for Short-Term Pain and Physical Performance Improvement in Elderly Patients with Knee Osteoarthritis: A Prospective Four-Case Reports

**DOI:** 10.3390/geriatrics10060170

**Published:** 2025-12-18

**Authors:** Rafaela F. Rodrigues, Carlos Marcelo de Barros, André A. V. Lima, Felipe T. Vilela, Vanessa B. Boralli

**Affiliations:** 1 Faculty of Pharmaceutical Sciences, Federal University of Alfenas, Alfenas 37130-001, Brazil; andre.lima@sou.unifal-mg.edu.br; 2 Faculty of Medicine, Federal University of Alfenas, Alfenas 37130-001, Brazil; carlos.barros@unifal-mg.edu.br (C.M.d.B.); felipe.vilela@sou.unifal-mg.edu.br (F.T.V.)

**Keywords:** joint disease, sensory nerve denervation, cooled radiofrequency, case series, elderly

## Abstract

Background and Objectives: Osteoarthritis (OA) is a common cause of chronic pain. In refractory cases, cooled radiofrequency (CRF) of the genicular nerves is indicated. However, recent studies suggest that traditional targets may be insufficient, proposing the inclusion of the recurrent fibular nerve and the infrapatellar branch of the saphenous nerve. This study reports a prospective four-case series evaluating short-term outcomes of CRF at five revised targets in elderly Brazilian patients. Case Report: The study (CAAE No.: 55647722.5.0000.5142) included four patients (three women, one man; mean age 73.8 years) with moderate to severe refractory knee pain underwent diagnostic nerve block followed by ultrasound-guided CRF. After 30 days, three patients reported pain reduction, including two who experienced substantial improvement. One patient maintained severe pain. Improvements in physical performance, knee flexion, and extension were observed in patients who responded clinically, while individuals with coexisting myofascial pain showed limited functional gains. One patient experienced mild transient pruritus. In this prospective case series, CRF applied to five revised targets appeared feasible and well tolerated, with short-term improvement in pain and function in some patients. These preliminary descriptive findings support further investigation in larger controlled studies.

## 1. Introduction

Osteoarthritis (OA) is a chronic, degenerative, and complex pathology that involves the joint as a whole and encompasses structural alterations of the hyaline articular cartilage, subchondral bone, ligaments, capsule, synovium, and periarticular muscles [1,2]. The prevalence in Brazil is 33% of the population (approximately 40 million Brazilians) [3].

The incidence of knee OA may increase due to the following risk factors: age (elderly), female gender, obesity, previous knee injuries, knee malalignment, knee extensor muscle weakness, heavy activities involving frequent squatting and heavy lifting, high-impact sports, and genetic factors [4,5,6,7,8].

Generally, the diagnosis of OA is made through a combination of clinical analysis based on symptoms (such as pain, brief morning stiffness, and functional limitations) and physical examination (including crepitus, restricted or painful movement, joint tenderness, and bone enlargement). In addition, imaging tests (X-rays or magnetic resonance) may be used to classify the degree of OA using radiological scales, such as the Ahlbäck scale, which ranges from 0 to 4, with 0 being normal, 1 with joint space narrowing less than 3 mm of the joint space or less than 50% in the other compartment (with or without subchondral sclerosis), 2 with joint space obliteration, 3 with bone defect or loss less than 5 mm, and 4 with bone defect and/or loss of 5 to 10 mm [9,10].

Pain in the knee due to OA is the most frequent symptom and is regularly classified as intermittent, and in most cases, of a mechanical nature. As it becomes more intense or frequent, patients usually classify their suffering as intolerable [11].

There are, therefore, few options for patients with long-lasting painful symptoms, refractory to conservative treatment, who are not eligible for arthroplasty surgery due to coexisting medical conditions or comorbidities, or for those who do not want surgery or wish to postpone arthroplasty. The options include blocking the sensory nerves of the knee with corticosteroids and local anesthetics or ablating the sensory nerves using radiofrequency (RF). Both techniques aim to reduce pain perception by inhibiting the sensory nerves of the knee [12,13].

The classic cooled radiofrequency (CRF) technique involves denervation at three targets, performed on the genicular nerves: superomedial, superolateral, and inferomedial, and uses these targets as a strategic option for obtaining analgesia in the knee [14]. Several studies have been conducted and demonstrated the effectiveness of this technique for relieving joint pain in the knee [15,16]. Based on new anatomical studies in cadavers, the classic therapeutic three-targets for CRF are incomplete and imprecise. Thus, revised therapeutic targets were proposed, encompassing denervation in five targets: the genicular nerves (superomedial, superolateral, and inferomedial), the recurrent fibular nerve, and the infrapatellar branch of the saphenous nerve [17,18,19,20,21,22]. Proponents of the revised five-target CRF technique conducted a study comparing nerve blockade, classical three-target CRF, and revised five-target CRF, and the latter proved to be effective in significantly reducing pain when compared to other techniques [23].

Since age is a risk factor for OA and the gold standard treatment (total arthroplasty) may not be feasible for this population due to the high surgical risk associated with comorbidities, minimally invasive techniques, such as cooled radiofrequency, are gaining relevance because they provide analgesia and functional improvement with a low complication rate, directly impacting the quality of life of this population [24,25]. It is known that Brazil is undergoing a demographic transition, with projections that 20% of the population will be elderly by 2050 [26,27]. Thus, testing minimally invasive techniques represents an alternative adapted to the reality of the country.

To the best of our knowledge, the revised five-targets have never been tested in the Brazilian elderly population. Therefore, the study hypothesis was that the revised five-target CRF could be a good option for older adult patients with refractory OA. Thus, the objective of this prospective four-case study was to evaluate for the first time the short-term efficacy of this minimally invasive procedure in the elderly Brazilian population.

## 2. Methods

### 2.1. Ethical Concerns

This was a prospective four-case study, authorized by the Ethics Committee of the Federal University of Alfenas (UNIFAL-MG), CAAE No. 55647722.5.0000.5142. The screening, performance of the procedure, and follow-up of patients took place at the Sinpain Alfenas Clinic. All patients were invited to participate, informed about the procedure, and informed that they could withdraw their consent at any time. All participants signed the Free and Informed Consent Form for their participation in the research and the anonymous dissemination of the results.

### 2.2. Participants

The inclusion criteria were: patients of both sexes, aged 60 years or older, with unilateral or bilateral knee osteoarthritis, with confirmed medical diagnosis (radiography and magnetic resonance imaging, Alhbäck classification 2 or 3), with chronic refractory pain, of moderate or severe intensity (score ≥ 5 on the visual analogue scale—VAS), for at least 3 months, who obtained relief of at least 50% of pain after ultrasound-guided blockade test with lidocaine (1%) in the genicular nerves.

The exclusion criteria were: patients under 60 years of age, even if diagnosed; patients not diagnosed through imaging tests with OA; patients who had undergone previous knee surgery in the last 3 months before inclusion; patients who had received intra-articular corticosteroid injections in the last 3 months before inclusion; patients with uncontrolled neurological or psychiatric diseases; patients with uncontrolled diabetes; pregnant patients; patients with cancer; patients with lumbar radiculopathy; patients with rheumatic disease; patients on continuous anticoagulant therapy; patients on continuous use of opioids; patients who could not understand verbal or written language.

**Figure 1:**
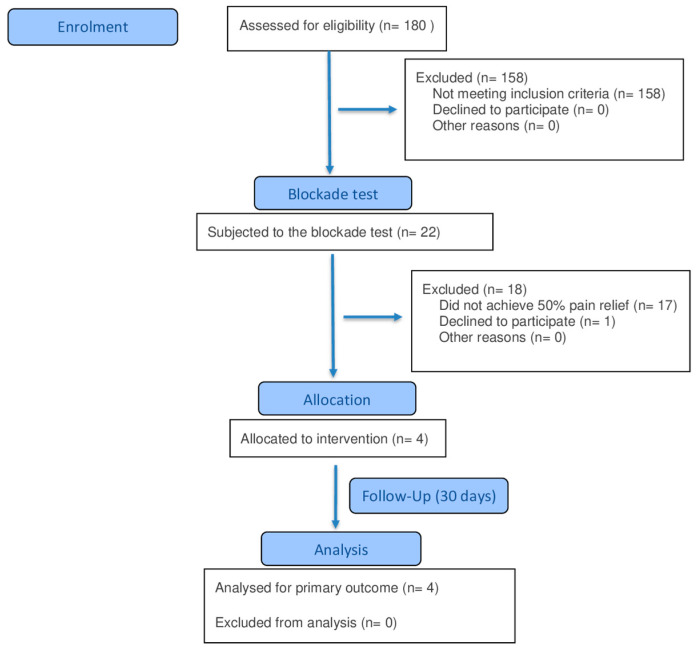
Flow chart of patient screening.

Since this is a prospective study, it is important to show the flow chart for patient screening (Figure 1).

### 2.3. Therapeutic Intervention

As an internal protocol at the Sinpain Alfenas Clinic, all patients with OA are prescribed pregabalin (75 mg, twice daily, orally, Viatris, Pittsburgh, PA, USA) and duloxetine (60 mg/day, orally, EMS, Brazil) for continuous use, starting 14 days before the blockade test to achieve steady-state, and patients continued to use the medications (without changing doses) throughout the follow-up period. After discharge from the hospital, dypirone (1 g, orally, up to 4 g/day, when necessary, Sanofi, Brazil) and tramadol (50 mg orally, 6/6 h, when necessary, Grunenthal do Brasil, Brazil) were prescribed as rescue medication. In addition, the participants were advised to seek medical attention if the case of pain did not subside with the use of rescue medications during the follow-up period.

All patients participating in the study underwent a block test in the 3 genicular nerves, using 2 mL of 1% lidocaine (Cristália, Brazil), guided by ultrasound, before the CRF procedure, which took place at the Sinpain Alfenas Clinic. Patients who achieved an improvement of at least 50% with the block test underwent CRF, 7 to 30 days after the block test (time needed for the Brazilian Unified Health System (SUS) to authorize the procedure). Each patient underwent CRF only once.

The procedure was performed with aseptic technique in the surgical center of the Santa Casa de Alfenas. Patients were monitored for blood pressure, electrical cardiac activity (by cardioscope), and arterial oxygen saturation. Anesthesia was performed under light sedation, using 2% lidocaine for local anesthesia, and total venous sedation with target-controlled infusion of propofol (25 to 50 mcg/Kg/min, Cristália, Brazil) and remifentanil (0.025 to 0.05 mcg/Kg/min, Cristália, Brazil), until Ramsay 2 sedation was obtained, accompanied by an anesthesiologist throughout the procedure.

Then, patients were positioned on a fluoroscopy table in a supine position, with a cushion under the knee (in the popliteal fossa) to alleviate discomfort and place the knee in a slightly flexed position. The skin and subcutaneous tissues superficial to the target nerves were anesthetized with 2 mL of 1% lidocaine under sterile conditions. Patients underwent motor blockade testing before the CRF itself to ensure that no motor structures were affected. For this test, 45 volts and 2 hertz in a cooled RF generator (COOLIEF^®^ Avanos, Alpharetta, GA, USA) were used. In the event of a negative result in the motor test, the CRF procedure was followed, using 75 mm needles with a 4 mm active tip (COOLIEF^®^ Avanos, Alpharetta, GA, USA) guided by fluoroscopy. The same cooled RF generator was used, applying 45 volts and 2 hertz, at a temperature of 60 °C, and applied for 150 s. It is important to highlight that the Coolief^®^ system features automatic internal cooling through water circulation, keeping the electrode tip cooled to approximately 4 °C, as specified by the manufacturer. The equipment was used in accordance with technical recommendations and underwent a functional check before each intervention, ensuring the calibration and safety of the parameters used.

**Figure 2:**
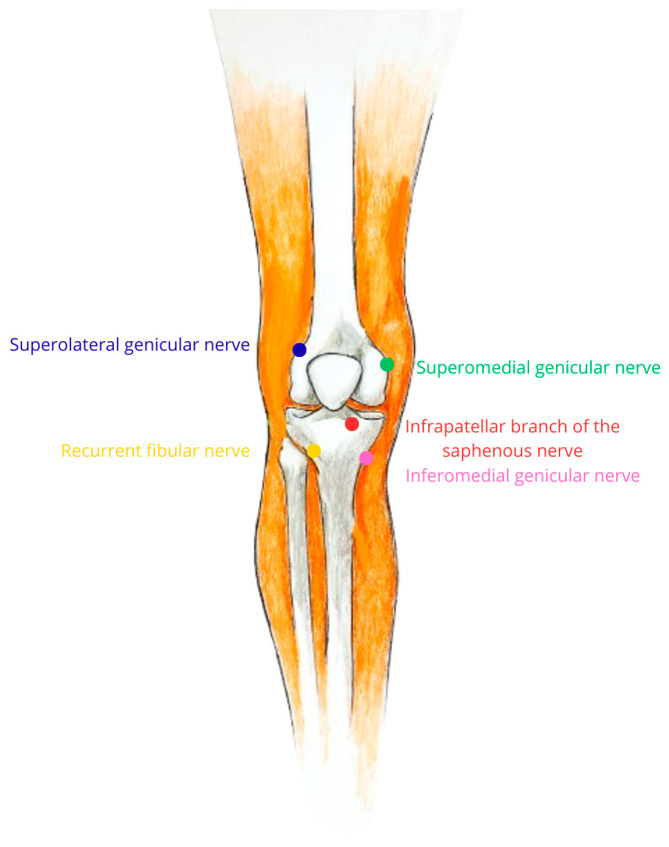
Schematic image of the five revised nerves for cooled radiofrequency (CRF). The blue circle (upper left corner) indicates the insertion point of the system for denervation of the superolateral genicular nerve. The green circle (upper right corner) indicates the insertion point of the system for denervation of the superomedial genicular nerve. The red circle (in the middle, near the patella) indicates the insertion point of the system for denervation of the infrapatellar cranch of the saphenous nerve. The yellow circle (lower left corner) indicates the insertion point of the system for denervation of the recurrent fibular nerve. The pink circle (lower right corner) indicates the insertion point of the system for denervation of the inferomedial genicular nerve.

The targets were the superomedial genicular nerve, the superolateral genicular nerve, the inferomedial genicular nerve, the recurrent fibular nerve, and the infrapatellar branch of the saphenous nerve (Figure 2). The targets were located as described by the reference authors [23]. Briefly, the target for the superomedial genicular nerve was located at the junction of the medial femoral shaft and condyle in the anterior–posterior (A-P) view, and in the true lateral view, it was in the middle of the femur. In the A-P view, the target location for the superolateral genicular nerve was found at the junction of the lateral femoral shaft and condyle, and in the true lateral view, it was at the midpoint of the femur. In the A-P view, the inferomedial genicular nerve had its target location at the junction of the medial tibial shaft and tibial condyle, while in the lateral view, it had its target location in the middle of the tibia. For the recurrent fibular nerve, a longitudinal line was drawn below Gerdy’s tubercle and 1 cm below the inferior border of the tubercle, and the needle was inserted until the tip touched the bone. For the infrapatellar branch of the saphenous nerve, a longitudinal line (target line) was drawn 4 cm medial to the apex of the patella, and two transverse lines were drawn passing through the apex of the patella and the top of the tibial tuberosity. The needle was inserted longitudinally at the proximal end of the target line and advanced to its distal end, deep into the subcutaneous tissue. It is not usually necessary to use imaging to identify the recurrent fibular nerve and the infrapatellar branch of the saphenous nerve, but in cases where anatomical changes were observed that could negatively impact the procedure, ultrasound could be used to aid in confirmation. For these four patients, it was not necessary.

The surgical procedure and post-procedure protocol followed the Brazilian Ministry of Health’s safe surgery protocol [28]. After the procedure, the patients went to anesthetic recovery for two hours, under monitoring, were taken to their rooms, and discharged the following day.

### 2.4. Data Monitoring

Pain intensity was assessed using the Visual Analog Scale (VAS) before and 30 days after CRF, at the Sinpain Alfenas Clinic. Time to up and go (assessment of physical performance); goniometry (assessment of range of motion, active extension and active flexion), palpation of trigger points (quadriceps, Iliotibial tract, Medial gastrocnemius, and Hamstrings), and shortening and flexibility tests were also carried out in person at the Sinpain Alfenas Clinic, before and 30 days after CRF. All variables will be reported only for the knee undergoing CRF.

The 30 days were defined as representing the most feasible interval for monitoring, ensuring methodological consistency and feasibility in data collection.

Potential confounding factors, such as variability in pain perception (VAS), concomitant use of medications, comorbidities that affect functional performance (TUG), as well as differences in assessment techniques (goniometry and trigger point palpation), were considered through standardization of procedures, reinforcing that the tests were administered by physicians specializing in pain, and the medications used were recorded. In addition, the potential confounding factors as comorbidities, are an inherent limitation in the context of some patients with OA.

Data regarding age, sex, weight, height, body mass index, comorbidities, and medications of continuous use were obtained through anamnesis during the appointment on the day of the blockade test, held at the Sinpain Alfenas Clinic. Data regarding the knee where the procedure was performed were obtained through records in electronic medical records.

Patients were encouraged to report to the researchers the occurrence of adverse effects at any time after CRF. Contact with the researchers could be made by phone, app of text messages, or email, which was provided to patients for any clarifications or reports during the monitoring period. All clinical and functional outcomes—including Visual Analog Scale (VAS) pain scores, time to up-and-go test, active knee extension and flexion, trigger point assessment, and flexibility tests—were assessed before and 30 days after CRF. Results are presented using descriptive statistics (individual values, means, and ranges), appropriate for a four-case prospective study. No inferential statistical tests were performed, as the sample size does not allow valid interpretation beyond descriptive comparison.

## 3. Study Cases Presentation

Four elderly patients with refractory knee OA underwent CRF using five revised anatomical targets. Individual sociodemographic and clinical characteristics are presented in Table 1.

Only 2 patients (50%) had comorbidities (patients C and D), both of whom had mental illness (depression), and one of the patients (patient D) also had metabolic disorders (diabetes, hypercholesterolemia, and arterial hypertension). Both are being monitored by specialists and are undergoing pharmacological treatment (for depression, one patient was using sertraline 100 mg, once a day, orally, continuously, and the other patient was using nortriptyline 50 mg, twice a day, orally, continuously. For metabolic disorders, the patient was using rosuvastatin 20 mg a day, orally, continuous use for hypercholesterolemia; metformin 500 mg, 3 times a day, orally, continuous use for diabetes, and losartan 100 mg a day, orally, continuous use for hypertension.

At the blockade test consultation, three of the four patients (75%) reported using dipyrone 1 g (orally) without a prescription in moments of intense pain, but with little improvement. In addition, two patients also reported using muscle relaxants without a prescription (caffeine 30 mg + carisoprodol 125 mg + diclofenac sodium 50 mg + paracetamol 300 mg, orally) or nonsteroidal anti-inflammatory drugs (etoricoxib 120 mg, orally) in moments of pain exacerbation, with reports of improvement only with the use of nonsteroidal anti-inflammatory drugs.

Regarding the procedure and the post-procedure observation period, no pain medication was needed after the procedure until the patient was discharged from the hospital. Adverse effects resulting from the procedure occurred in only 1 patient (25%, patient B), who presented mild pruritus (that was assessed by physical examination and medical history) on the procedure leg from the knee down, which began immediately after the procedure. No motor deficits, infection, or sensory disturbance were observed. This patient was prescribed two doses of loratadine 10 mg orally, with 12 h intervals between doses and observation. After 1 h of the first dose, the pruritus disappeared. The patient remained under observation for the same time as the other patients (2 h) and was discharged from the hospital the following day without complications.

Only patients B and C reported that it was necessary to use rescue medication more than 4 times during the follow-up period. The patients also reported that the medications were necessary starting 1 week after CRF. Only one patient (patient C) needed to seek medical assistance (emergency room) due to severe pain 20 days after CRF. This patient was prescribed ibuprofen (600 mg, orally, four times a day for five days) and paracetamol (750 mg, orally, up to four times a day, as needed). This prescription was not made by the doctors on the team conducting this study, but rather by the doctors on duty. We emphasize that follow-up by the study team was available, but the patient chose to go to the emergency room.

**Figure 3:**
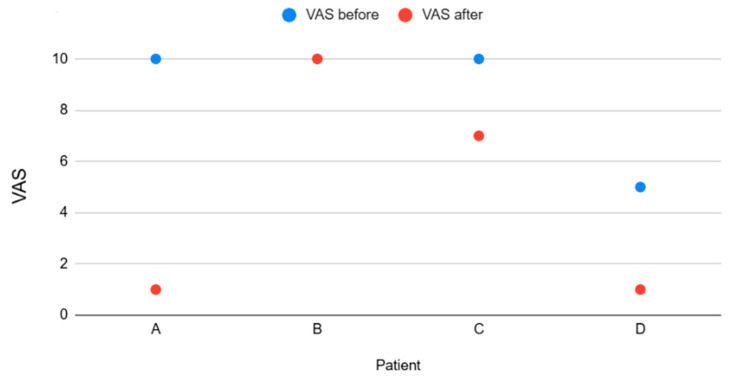
Visual Analogic Scale (VAS) heterogeneity of patients before and after the cooled radiofrequency (CRF).

Thirty days after the procedure, three patients (A, C, and D) reported reduced pain intensity compared with baseline. Two of these patients (A and D) experienced marked improvement, with VAS decreasing from severe or moderate levels to mild pain. Patient C showed partial improvement. One patient (B) reported no change in pain intensity and maintained severe pain. Mean VAS decreased from 8.75 at baseline to 4.75 after CRF, reflecting heterogeneous but generally favorable individual responses. The VAS details before and after denervation, for each patient, are described in Table 2 and in Figure 3.

**Figure 4:**
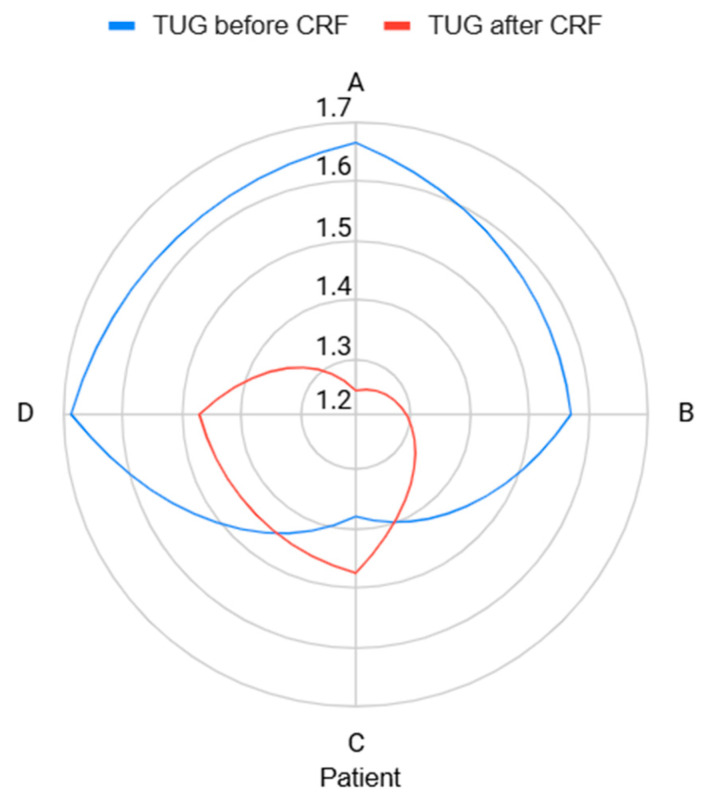
Radar chart of the time to up and go test before and after the cooled radiofrequency (CRF).

Before CRF, all four patients demonstrated impaired performance, with TUG times ≥10 s. After 30 days, patients who reported pain relief (A and D) also showed notable improvement in TUG performance. Patients B and C, who exhibited persistent or partially improved pain, showed little to no functional gain. The mean execution time decreased from 24.5 s to 20.25 s, consistent with the individual clinical trajectories. The TUG details before and after denervation, for each patient, are described in Table 3 and in Figure 4. 

**Figure 5:**
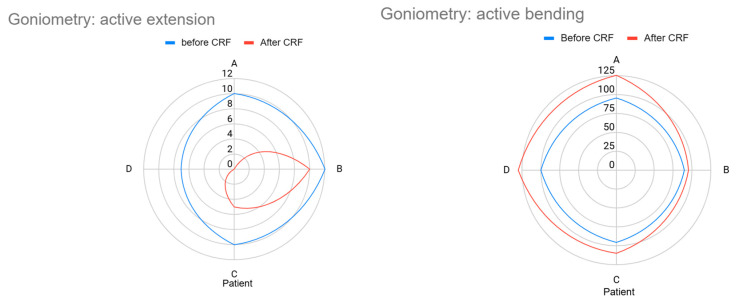
Radar chart of the goniometry test before and after the cooled radiofrequency (CRF).

Table 4 and Figure 5 present the goniometry results for each patient. Patients A and D demonstrated clear improvement in both active extension and flexion, with restoration of full extension and flexion gains exceeding 20 degrees. Patients B and C showed minimal improvement, which aligned with their lack of pain remission. On average, patients improved knee flexion from 95° to 115° and reduced extension limitation from 9.75° to 3.75°.

Muscle palpation and flexibility testing (Table 5) revealed improvement in myofascial trigger points and shortening in patients A and D. In contrast, patients B and C exhibited new or persistent myofascial sensitization, suggesting an alternative or concomitant mechanism contributing to their pain. These findings were confirmed through clinical re-evaluation and guided their subsequent referral for physical therapy.

**Figure 6:**
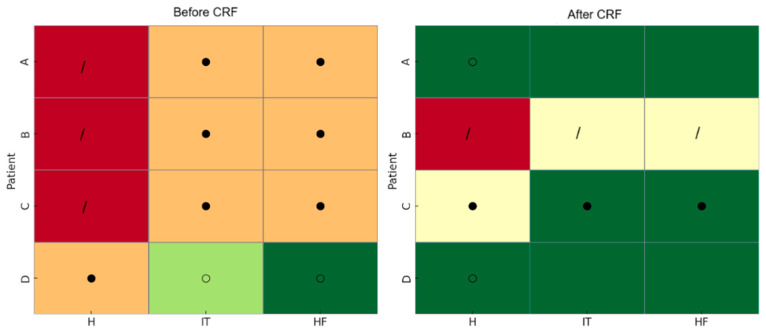
Flexibility and tightness test. Colors indicate the degree of tightness/flexibility (dark green = normal, light green = normal, but tending towards shortening, orange = moderate shortening, light yellow = moderate shortening, but less shortening than orange, red = severe tightness). Symbols represent pain: ○ mild, ● moderate, / severe. Muscles: H = hamstrings, IT = iliotibial band, HF = hip flexors).

In the assessment of flexibility and muscle shortening, a heterogeneous response was observed among patients. Patient A showed global improvement, going from marked shortening and intense pain to mild shortening with minimal or no pain, reflecting significant functional gain. Patient D also demonstrated significant improvement, with normalization of flexibility and elimination of pain. In contrast, patients B and C showed unfavorable or limited progression. Patient B developed worsening myofascial pain and maintained shortening, while patient C showed only partial improvement, still with moderate shortening and persistent pain (Figure 6).

From the results of the functional tests, it was possible to observe that there was no or incomplete pain relief in patients B and C 30 days after CRF. Therefore, these patients underwent a new medical evaluation, and it was confirmed through imaging exams that there was no excessive nerve damage; that is, the maintenance of pain intensity or partial pain relief did not occur as a result of the injury caused by CRF. So, they were clinically co-diagnosed with myofascial pain syndrome using the diagnostic criteria proposed by Simons et al. [29], considering their adaptations over time [30,31]. The management of myofascial pain syndrome in these patients involved medical monitoring and referral to physical therapy (to release trigger points, stretching, and analgesic physical therapy—Transcutaneous Electrical Nerve Stimulation, laser, and ultrasound). The patients continued to receive medical and physical therapy follow-up after the co-diagnosed with myofascial pain. However, as follow-up beyond 30 days is outside the scope of this study, we did not have access to the medical records regarding the continued management of these patients.

## 4. Discussion

In the present case study, there was a predominance of female patients, over 60 years of age, and suffering from obesity. This observation is in line with the risk factors for osteoarthritis already described in the literature [4,5,6,7,8,32,33,34,35,36,37,38].

Regarding the comorbidities observed in the present study, metabolic disorder is highly related to obesity, further increasing the risk for developing OA [4,5,6,7,8]. However, it is not possible to establish direct causality in these patients since we did not know them previously. In addition, depression is very common in patients with chronic pain, and its coexistence tends to aggravate both disorders. However, the correlation between depression and chronic pain is still uncertain. Several hypotheses have been raised (such as a decrease in the bioavailability of monoamines, production of Brain-Derived Neurotrophic Factor, production of inflammatory factors, and an increase in excitatory glutamatergic receptors) and remain under investigation [39]. Again, we cannot establish whether the pain came first and triggered the depression or whether it was the other way around, as we did not know the patients before the study.

These comorbidities can significantly influence pain perception and response to radiofrequency denervation. Depression has been linked to descending inhibitory circuit malfunction and central sensitization, as well as catastrophizing and worse coping, all of which can enhance the unpleasant experience and shorten the duration of recovery. Metabolic illnesses, such as diabetes and obesity, are characterized by low-grade chronic inflammation and, in some circumstances, peripheral neuropathy, both of which lead to increased nociceptive sensitization and a diverse response to intervention. Furthermore, obesity can make it harder to properly position the needles, reducing the procedure’s efficiency. Thus, these disorders constitute potential confounding factors, emphasizing the importance of multimodal care and cautious results interpretation [40,41,42].

Because OA presents a broad symptom complex, affected individuals end up resorting to self-medication, mainly analgesics and non-steroidal anti-inflammatory drugs, as the most accessible and practical method for managing painful symptoms. This can generate risks for the individual, such as poisoning and adverse reactions, but also for the health system, generating unnecessary costs, in addition to delaying diagnosis and treatment [43]. A study conducted by Brazilian researchers [43] showed that more than 60% of participants with OA self-medicate. Another survey of users of SUS showed that 76% of participants had self-medicated in the month before the survey, with a predominance of “other analgesics and antipyretics” (3rd ATC level) and Losartan (5th ATC level) [44]. This high prevalence of self-medication was also observed in the present study since all patients reported self-medicating when their pain worsened. This shows that the irrational use of medicines continues to be a public health problem, as it raises concerns about therapeutic efficacy and possible drug intoxication [45].

Self-medication will hardly ever stop, but when carried out responsibly and under the supervision of a specialist, it can contribute to the rational use of medicines. In this context, the pharmacist is fundamental, as they are responsible for guiding the patient during dispensing, reducing side effects, disguising illnesses, and combating drug resistance. Pharmaceutical care involves identifying and solving problems related to medicines, promoting safer, more accessible, and effective therapeutic options. In addition, they aim to educate users, ensuring that the pharmacy is seen as a point of health care and not as a business. This preventive action also contributes to reducing the costs of hospitalizations and curative treatments in the SUS, reinforcing the pharmacist’s responsibility for promoting public health and the rational use of medicines [45].

Patients in the present study received guidance as part of their pharmacological education, informing them that some medications used in self-medication are not indicated for OA pain [46]. This health education contributes to the reduction in medication-related problems, contributing to the World Health Organization’s 3rd global challenge, “Medication Without Harm”, with the aim of patient safety [47]. It is also in line with the Organization of United Nations (ONU) 2030 Agenda for Sustainable Development, specifically within the scope of three global goals: “Health and Well-being” (SDG3), “Quality Education” (SDG4), and “Responsible Consumption and Production” (SDG12) [48].

It is important to note that the use of pregabalin + duloxetine as a clinical protocol at the Sinpain Alfenas clinic is evidence-based, since a large proportion of patients with OA experience pain sensitization through neuropathic mechanisms, which arise from structural changes in joint innervation or nerve alterations in the peripheral nervous system or spinal cord; or through central pain mechanisms, which include increased activity of descending pain facilitation pathways and loss of descending antinociceptive pathways [9]. However, it is worth noting that the use of this combination or other rescue medication by patients during the follow-up period may alter the pain trajectory, impacting the results obtained, which is an inherent limitation of real patients with chronic degenerative diseases such as OA.

Several studies available in the literature report that classical three-targeted CRF procedures have gained prominence in recent years for the management of knee OA pain in patients refractory to conservative treatment, as it is a minimally invasive technique that uses temperature to reach therapeutic targets and is safe and effective in the short and medium term [12,23,49,50], which is in line with what was observed in this study. In addition, the review carried out by Oladeji and Cook [51] included 8 studies that used CRF for the treatment of chronic knee OA and showed that CRF is an emerging ablative procedure with encouraging initial results; however, more long-term prospective clinical studies are needed to better characterize how CRF can be used to treat chronic knee pain, which is in line with what was observed in the present study.

The findings of the present study suggest that revised five-target cooled radiofrequency may contribute to improving pain, flexibility, and functional capacity in some patients with knee OA in the short term, and has also been shown to be safe, but this was not uniform in all cases, despite knowing the need for comparative studies to prove the efficacy and safety of the technique. In the same way that pain relief and function improved after expanded CRF was observed in the present study, the proponents of the revised five-targeted CRF technique also observed it [23]. In addition, another single-arm cohort study showed that expanded cooled radiofrequency targeting the genicular nerves, vastus medialis nerve, vastus lateralis nerve, and nervus intermedius resulted in long-term (>18 months) pain improvement and global improvement [52]. In this small case series, some patients experienced short-term improvement, which is consistent with observations from studies evaluating expanded techniques [53]. However, there are few clinical studies available in the literature on expanded cooled radiofrequency techniques and comparative studies between three-target techniques and expanded techniques, which show the importance of case studies like the present study.

Although CRF is a minimally invasive technique, it is not free from adverse effects resulting from the placement of devices in the targets and the application of radiofrequency. Thus, the adverse effect reported by a single patient in the present four-case study, because of the technique (mild itching, knee downwards, in the procedure leg), follows that reported in the literature and does not generate any major concern [49].

The present study also showed that the myofascial pain syndrome, present in two of the four patients, may have contributed to the continued pain intensity, since this syndrome may have been triggered as a result of protection against joint pain, which leads to compensatory overload, triggering muscle imbalance and biomechanical changes. It is known that the co-occurrence of different types of pain leads to chronic muscle load, which triggers the formation of myofascial trigger points in tense regions of the skeletal muscle and fascia, giving rise to myofascial pain syndrome [54]. Observational studies have demonstrated a positive correlation between knee osteoarthritis and myofascial trigger points in lower limb muscles such as vastus lateralis, rectus femoris, and gracilis [55,56]. However, it is still unknown why this co-occurring myofascial pain occurs only in some patients and not in others.

Finally, it is important to emphasize that OA is the 15th leading cause of years lived with disability (YLDs) globally, causing significant pain, activity limits, and decreased quality of life. OA also has a significant economic impact, accounting for both direct and indirect costs, with the average annual expense of OA for individuals worldwide ranging from $700 to $15,600 (2019 USD) [33]. Therefore, it is important that cost-effectiveness studies on CRF in five-revised targets and other interventional techniques are carried out, so that the trade-offs and uncertainties involved in choosing these different alternatives can be clarified. In this way, the best option for the management of refractory OA pain can be chosen, helping decision-makers to allocate scarce resources efficiently and effectively.

Regarding the patients’ perspective on the minimally invasive technique used for the management of knee OA, patients A and D reported significant improvement in pain, physical performance, and improved range of motion, which directly impacted their quality of life, as they reported that they were able to perform routine activities again, such as going to the supermarket, without feeling pain. Patient C reported that she had partial pain relief, which was better than before, but hoped that she could have improved a little more. Patient B reported that she did not feel any improvement after the procedure and was frustrated.

This study has some limitations that should be considered when interpreting the results. Because of the small sample size (n = 4), no inferential analysis is appropriate, and all findings must be interpreted descriptively. In addition, there may have been a possible selection bias due to the requirement of ≥50% relief in the diagnostic block before CRF, even though it is a very common prerequisite when talking about sensory ablation by cooled radiofrequency. Also, because the joint function assessment had to be performed by physicians, it was not possible to blind the professionals to the outcomes. However, the researchers who analyzed the results were blinded throughout the follow-up, and the blinding was only removed at the time of data analysis. Furthermore, the short follow-up period (1 month) may not be sufficient to capture the medium- and long-term effects of the intervention. The absence of a control group (classical targeted CRF) prevents direct and robust comparisons that would allow for inferring causality with greater certainty. Moreover, concomitant use of pregabalin + duloxetine (as clinical protocol) or nonsteroidal anti-inflammatory drugs or tramadol (rescue medication) may have altered the pain trajectory, but we emphasize that this is a real-world limitation of a series of cases involving patients with degenerative diseases.

For future perspectives, population-based conclusions and generalizations, it is essential to conduct randomized controlled clinical trials to validate the observed effects. Additionally, cost-effectiveness studies are recommended to measure the economic and social benefits of the intervention.

## 5. Final Considerations

Because this report describes only four cases, all observations are purely descriptive and cannot be generalized. The results obtained with the patients in this four-case study, using CRF applied to revised five-targets in the knee, suggest that the technique is feasible, well tolerated in the short term, and may provide short-term pain relief and function improvement for some patients. Importantly, this was the first time that the revised targets protocol was applied in Brazilian patients, indicating that, in these four cases, the procedure was feasible and well tolerated. This study also highlighted the value of combining pain and health education as part of a biopsychosocial strategy for patients with chronic pain.

Nevertheless, the preliminary nature of these findings must be emphasized. The small number of participants, the absence of a control group, the short follow-up, and the possible influence of concomitant medications and selection bias represent important limitations that restrict the generalizability of the results. Therefore, while these early observations illustrate the feasibility and short-term tolerability of the technique in this small case series, additional comparative and randomized studies with larger samples and longer follow-up are essential to confirm its efficacy and safety in the medium and long term, in addition to the need for cost-effectiveness studies before its routine adoption in clinical practice.

## Figures and Tables

**Table 1:** Sociodemographic data and functional status before and after cooled radiofrequency (CRF) of study patients.

Patient	Age (Year)	Gender	Comorbidity	Knee of Procedure	Functional Status Before CRF	Functional Status After CRF
A	64	Male	None	Left	Moderate to significant limitation	Good functional performance
B	84	Female	None	Left	Significant limitation	Significant functional limitation, limited improvement
C	72	Female	Depression	Right	Severe limitation	Moderate to significant limitation, with joint improvement, but still significant impact on mobility
D	75	Female	Depression, Metabolic disorder (diabetes, hypertension, hypercholesterolemia)	Right	Severe limitation	Good articulation, but functional limitation

**Table 2:** Visual Analog Scale (VAS) before and 30 days after cooled radiofrequency (CRF) on five revised targets.

Patient	VAS (Before)	Interpretation (Before)	VAS (After)	Interpretation (Before)
A	10	Worst pain possible	1	Mild pain
B	10	Worst pain possible	10	Worst pain possible
C	10	Worst pain possible	7	Very severe pain
D	5	Moderate pain	1	Mild pain

**Table 3:** Time to up and go test (TUG) before and 30 days after cooled radiofrequency (CRF) on five-revised targets.

Patient	TUG Before CRF	TUG After CRF
A	21.6 s	13.2 s
B	22.5 s	22.0 s
C	26.3 s	23.4 s
D	27.6 s	22.4 s

**Table 4:** Goniometry before and 30 days after cooled radiofrequency (CRF) on five-revised targets.

Patient	Goniometry			
	Before CRF		After CRF	
	Active Extension	Active Bending	Active Extension	Active Bending
A	10° (flexion)	95°	0°	125°
B	12° (flexion)	90°	10° (holds flexion)	95°
C	10° (flexion)	95°	5° (still limited)	110°
D	7° (light flexion)	100°	0°	130°

**Table 5:** Trigger points before and 30 days after cooled radiofrequency (CRF) on five revised targets. Palpated muscles: Q—quadriceps; IT—Iliotibial tract; MG: Medial gastrocnemius; H: Hamstrings. The number of asterisks (regardless of color) indicates the number of trigger points (e.g., *: 1 trigger point; **: 2 trigger points; ***: 3 trigger points). The color of the asterisk indicates the trigger point status: yellow asterisks (*) indicate latent trigger points, and red asterisks (*) indicate active trigger points. The minus sign (-) indicates negative for trigger points. The forward slash (/) indicates that the trigger point is light, and the open circle (°) indicates that the trigger point is painful with radiating pain.

Patient	Before CRF				After CRF			
	Palpated Muscles							
	Q	IT	MG	H	Q	IT	MG	H
A	*	-	-	-	-	-	-	-
B	*	-	-	-	***	**	*°	*
C	*	-	-	*	**	*	-	*
D	*/	-	-	*	-	-	-	*

## Data Availability

The original contributions presented in this study are included in the article. Further inquiries can be directed to the corresponding authors.

## Abbreviations

The following abbreviations are used in this manuscript:

A-P	anterior–posterior
CRF	cooled radiofrequency
OA	Osteoarthritis
RF	radiofrequency
SUS	Brazil’s Unified Health System
UNIFAL-MG	Federal University of Alfenas
USD	American dollar
VAS	Visual Analog Scale
YLDs	Years lived with disability
